# Shell morphology and color of the subtidal whelk *Buccinum undatum* exhibit fine‐scaled spatial patterns

**DOI:** 10.1002/ece3.4015

**Published:** 2018-04-10

**Authors:** Hildur Magnúsdóttir, Snæbjörn Pálsson, Kristen M. Westfall, Zophonías O. Jónsson, Erla Björk Örnólfsdóttir

**Affiliations:** ^1^ Faculty of Life and Environmental Sciences University of Iceland Reykjavík Iceland; ^2^ Department of Aquaculture and Fish Biology Hólar University College Sauðárkrókur Iceland; ^3^ Vör – Marine Research Centre in Breiðafjörður Ólafsvík Iceland; ^4^ Present address: Fisheries and Oceans Canada Pacific Biological Station Nanaimo BC Canada

**Keywords:** *Buccinum undatum*, connectivity, phenotypic variation, shell color, shell morphology, spatial patterns, subtidal gastropods, whelk

## Abstract

Geographical patterns in morphology can be the result of divergence among populations due to neutral or selective changes and/or phenotypic plasticity in response to different environments. Marine gastropods are ideal subjects on which to explore these patterns, by virtue of the remarkable intraspecific variation in life‐history traits and morphology often observed across relatively small spatial scales. The ubiquitous N‐Atlantic common whelk (*Buccinum undatum*) is well known for spatial variation in life‐history traits and morphology. Previous studies on genetic population structure have revealed that it exhibits significant differentiation across geographic distances. Within Breiðafjörður Bay, a large and shallow bay in W‐Iceland, genetic differentiation was demonstrated between whelks from sites separated by just 20 km. Here, we extended our previous studies on the common whelk in Breiðafjörður Bay by quantifying phenotypic variation in shell morphology and color throughout the Bay. We sought to test whether trait differentiation is dependent on geographic distance and/or environmental variability. Whelk in Breiðafjörður Bay displayed fine‐scale patterns of spatial variation in shape, thickness, and color diversity. Differentiation increased with increasing distance between populations, indicating that population connectivity is limited. Both shape and color varied along a gradient from the inner part of the bay in the east to the outer part in the west. Whelk shells in the innermost part of Breiðafjörður Bay were thick with an elongate shell, round aperture, and low color diversity, whereas in the outer part of the bay the shells were thinner, rounder, with a more elongate aperture and richer color diversity. Significant site‐specific difference in shell traits of the common whelk in correlation with environmental variables indicates the presence of local ecotypes and limited demographic connectivity.

## 1 INTRODUCTION

Clines in species’ phenotypic traits are an important part of ecological and evolutionary studies (Levin, 1992) and can be extremely useful in quantifying the degree of connectivity among populations and for delineating population boundaries (Jones, Srinivasan, & Almany, 2007; Kough, Cronin, Skubel, Belak, & Stoner, 2017; Leis, van Herwerden, & Patterson, 2011; Woods & Jonasson, 2017). Geographical patterns in morphology can result from direct environmental control of physiological processes and body shape (Vermeij, 1978), differential adaptation to variable surroundings (Pinkert, Brandl, & Zeuss, 2016; Reinecke et al., 2016), or, alternatively, from random changes in genetically distinct populations (Kimura & Maruyama, 1971). Molluscan shells exhibit a wide variety of easily measurable morphological traits that make them ideal candidates for exploring the mechanisms giving rise to geographical patterns in phenotypic variation (Vermeij, 1978).

Phenotypic diversity and spatial variation in gastropod shell morphology have been well studied in intertidal and limnetic systems (Bourdeau et al., 2015; Johannesson, 2015; Johannesson, Johannesson, & Butlin, 2016; Rolán‐Alvarez, Austin, & Boulding, 2015; Trussell & Etter, 2001; Williams, 2017). However, there is a noticeable paucity of knowledge on geographical patterns, population connectivity, and within‐species diversity in marine species (Conover, Clarke, Munch, & Wagner, 2006), particularly in deep sea (>200 m depth) habitats (Mengerink et al., 2014; Taylor & Roterman, 2017) but also in shallower, coastal seas. These coastal zones are home to many commercially harvested gastropods, for which spatial management and conservation strategies are hampered by a scarcity of data on population processes (Jones et al., 2007; Kough et al., 2017; Leis et al., 2011; Machkour‐M'Rabet, Cruz‐Medina, García‐De León, De Jesús‐Navarrete, & Hénaut, 2017; Woods & Jonasson, 2017). Moreover, marine molluscs with direct development (e.g. many species of benthic gastropods) often have limited dispersal capabilities compared to species with a pelagic larval stage – a situation that may reduce demographic and genetic connectivity (Behrens Yamada, 1987; Bell, 2008), although adult dispersal capacity can still play a role (Johannesson, 1988; Kyle & Boulding, 2000; Leis et al., 2011; Marko, 2004).

**Figure 1:**
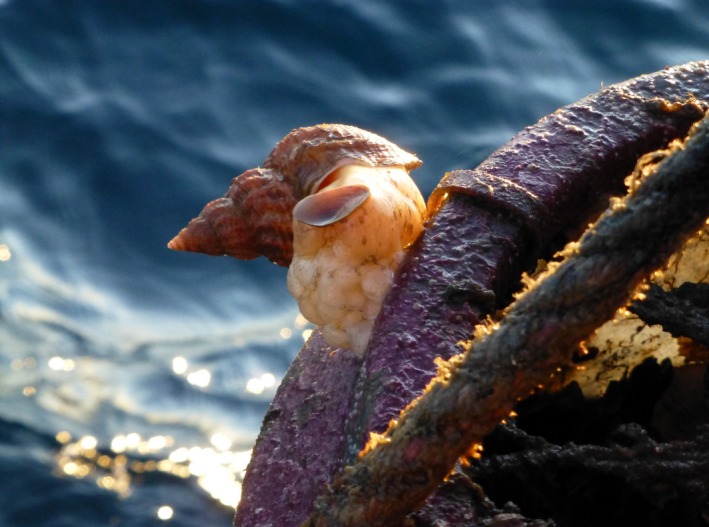
An adult female common whelk (*Buccinum undatum*) in Breiðafjörður Bay, Iceland (Photograph by Hildur Magnúsdóttir)

The common whelk (*Buccinum undatum*; Figure 1) is a commercially harvested subtidal (0–400 m) predator in the N‐Atlantic that is well known for its variable morphology, evident throughout the species’ distribution (Jeffries, 1867a,c; Kenchington & Glass, 1996; Magnúsdóttir, 2010; Mariani, Peijnenburg, & Weetman, 2012; Ten Hallers‐Tjabbes, 1979; Thomas & Himmelman, 1988). As early as the 1860's, malacologists had observed the difference between common whelk from shallow and deep areas (Jeffries, 1867b) and Golikov (1968) detailed how various morphological forms of the common whelk reflected the hydrological conditions of their habitat. The whelk's life‐history traits, such as direct development and limited adult dispersal may facilitate local adaptation and divergence of populations (Valentinsson, Sjödin, Jonsson, Nilsson, & Wheatley, 1999; Weetman, Hauser, Bayes, Ellis, & Shaw, 2006).

In more recent studies, sexual dimorphism has been demonstrated in populations on both sides of the N‐Atlantic, where females have on average higher and heavier shells (Kenchington & Glass, 1996; Ten Hallers‐Tjabbes, 1979). Thomas and Himmelman (1988) linked increased shell thickness and elongated apertures of Canadian common whelk with lobster and crab predation, and phenotypic differentiation in shell morphology of common whelk around Ireland appears to be driven by environmental variation (Mariani et al., 2012). Genetic analysis of migration trends in three locations in the UK indicated an inshore–offshore migration of whelk, which could be linked to inshore–offshore gradients in environmental variables (Weetman et al., 2006).

**Figure 2:**
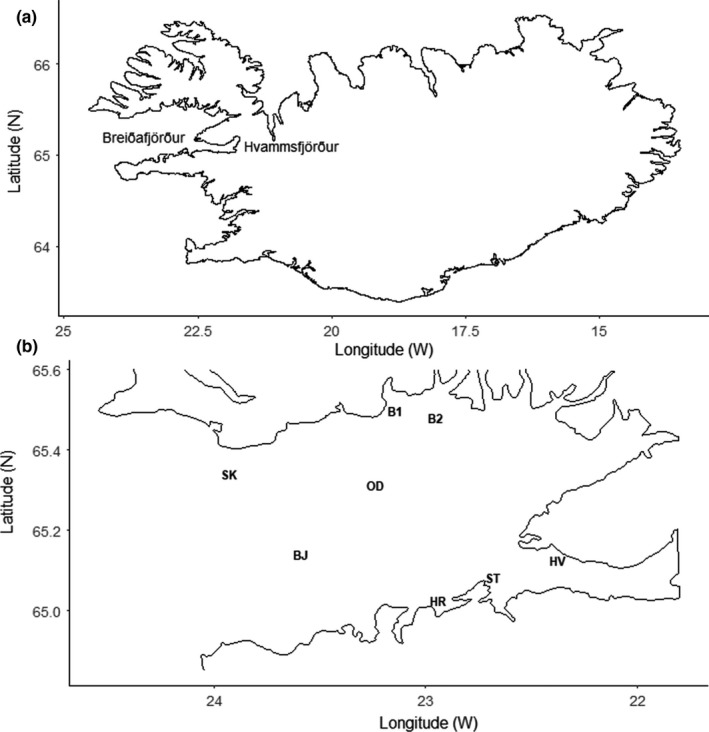
(a) Breiðafjörður and its tributary fjord, Hvammsfjörður, in W‐Iceland. (b) Common whelk (*Buccinum undatum*) sampling sites in Breiðafjörður Bay, W‐Iceland. Distance between the two closest sites, B1 and B2, is 9 km and the distance between the two furthest sites, HV and SK, is 76 km

In Breiðafjörður Bay, a wide and shallow bay in W‐Iceland with an indented coastline and a large number of islands and skerries (Figure 2a), the common whelk exhibits striking polymorphism in shell traits such as color and shape (Magnúsdóttir, 2010). Our previous work (Pálsson, Magnúsdóttir, Reynisdóttir, Jónsson, & Örnólfsdóttir, 2014) revealed that genetic differentiation in mitochondrial and microsatellite markers increased with geographic distance and that populations separated by 20–30 km within the bay were significantly different, reflecting limited dispersal. Similarly, Weetman et al. (2006) and Mariani et al. (2012) observed that genetic differentiation of the common whelk increased with geographic distance.

Here, we extend our previous study (Pálsson et al., 2014) to evaluate whether the common whelk in Breiðafjörður Bay is composed of different populations based on phenotypic variation in shell morphology and color, and whether the differentiation between populations is dependent on geographic distance and/or environmental variability. The shell shape and color of whelk within Breiðafjörður Bay was quantified and the association with depth and substrate type analyzed. To evaluate these effects whelk were sampled on a gradient along the bay from the inner part in the east to the outer part in the west. Shell shape was measured using both geometric and traditional morphometrics, and color was measured using categorical scoring.

## 2 MATERIALS AND METHODS

### 2.1 Sampling

Adult common whelk (*Buccinum undatum*; Figure 1) were collected using baited traps at eight locations in Breiðafjörður Bay in western Iceland in 2014, separated by 9–76 km (Figure 2b, Table 1). One sample (HV) came from the tributary fjord Hvammsfjörður (Figure 2a,b). In total, 344 individuals were obtained, 18–68 from each site. The whelks were gently removed from the shell with forceps for identification of sex after which the shells were cleaned and stored for morphometric and color analysis. All individuals were included in the color analysis while those with a broken aperture lip were excluded from the shape analysis (Table 2). The proportion of decollated shells (i.e., protoconch and 1–2 whorls missing) at each site was noted (Table 2) and while decollated shells were not excluded from the analysis, we took that variable into account in the partitioning of the shape variation.

**Table 1:** Summary of the spatial and environmental variables of the sample sites of common whelk in Breiðafjörður Bay. The sites are arranged in descending order from east to west

Location			Environment		
Site	Latitude (N)	Longitude (W)	Depth (m)	Substrate	Particle size
Hvammsfjörður (HV)	65.13	22.38	15	Hard	4
Stykkishólmur (ST)	65.08	22.68	18	Hard	4
Hrútey (HR)	65.03	22.94	36	Sand	2
Brjánslækur 2 (B2)	65.48	22.95	37	Mud	1
Brjánslækur 1 (B1)	65.50	23.14	37	Mud	1
Oddbjarnarsker (OD)	65.31	23.23	43	Mud	1
Bjarneyjaráll (BJ)	65.14	23.58	125	Mud	1
Skor (SK)	65.34	23.92	53	Gravel	3

**Table 2:** Descriptive statistics of the common whelk collected at the sample sites in Breiðafjörður bay. The sites are arranged in descending order from east to west

Color					Shape mean ± *SE*								
Site	*N*	Color types	Striped	Shannon diversity	*N*	Male (prop)	Decollated apex (prop)	Shell height (mm)	Shell width (mm)	Aperture height (mm)	Aperture width (mm)	Shell weight (g)	Thickness
HV	40	8	0.13	1.46	8	0.25	0.50	63.61 ± 1.65	35.18 ± 0.84	30.59 ± 0.65	15.00 ± 0.33	13.36 ± 1.14	0.20 ± 0.009
ST	30	11	0.07	1.94	23	0.22	0.35	82.01 ± 0.32	45.47 ± 0.16	41.97 ± 0.21	19.70 ± 0.08	25.14 ± 0.25	0.30 ± 0.002
HR	54	7	0.17	1.91	19	0.37	0.21	69.90 ± 0.62	39.05 ± 0.34	34.77 ± 0.31	17.73 ± 0.15	16.40 ± 0.32	0.23 ± 0.003
B2	65	9	0.08	1.71	24	0.42	0.25	62.55 ± 0.51	35.90 ± 0.33	30.77 ± 0.28	16.22 ± 0.14	11.45 ± 0.22	0.17 ± 0.002
B1	51	15	0.22	2.28	28	0.32	0.11	72.44 ± 0.30	41.74 ± 0.17	35.99 ± 0.15	18.24 ± 0.08	16.89 ± 0.17	0.23 ± 0.002
OD	68	17	0.41	1.97	27	0.30	0.15	51.66 ± 0.23	28.80 ± 0.12	25.75 ± 0.11	12.53 ± 0.06	6.35 ± 0.09	0.12 ± 0.001
BJ	18	7	0.44	1.83	15	0.53	0.20	40.80 ± 0.24	23.06 ± 0.13	21.58 ± 0.10	9.87 ± 0.06	3.24 ± 0.08	0.08 ± 0.002
SK	18	9	0.28	1.91	9	0.22	0.67	54.78 ± 0.93	30.45 ± 0.58	28.49 ± 0.47	12.60 ± 0.21	13.60 ± 0.80	0.23 ± 0.001

### 2.2 Environmental variables

Depth and substrate type at each site were recorded (Table 1). The substrate was ranked from 1 to 4 according to average particle size, ranging from small particles (mud) to rocky substrate. Geographic distances between sites were calculated based on latitude and longitude using the *geosphere* package (Hijmans, 2016) in R (R Core Team 2016).

### 2.3 Traditional and geometric morphometrics

The shapes of 153 whelk larger than 35 mm (Table 2), considered to be sexually mature or close to sexual maturity (Magnúsdóttir, 2010), were analyzed. Sample size for the morphological analyses ranged from 9 to 28 individuals (Table 2) per site. Both traditional and geometric morphometrics were used to analyze the shell shapes (Hollander, Lindegarth, & Johannesson, 2005; Mariani et al., 2012; Thomas & Himmelman, 1988) to determine whether the two methods were in agreement and to allow comparison with previous studies on the common whelk that have used traditional morphometrics (Hollyman, 2017; Thomas & Himmelman, 1988).

#### 2.3.1 Traditional morphometrics

Height and width of shell and aperture were measured using digital vernier calipers to the nearest 0.01 mm. Shells were weighed to the nearest 0.01 g. The ratio between shell height and shell width was used as a general indicator of shell shape, that is, elongate versus rotund shells. The ratio between aperture height and width was similarly used as an indicator of aperture shape. Shell thickness was summarized by the ratio of the square root of the shell weight to shell height, which showed a linear relationship.

#### 2.3.2 Geometric morphometrics

**Figure 3:**
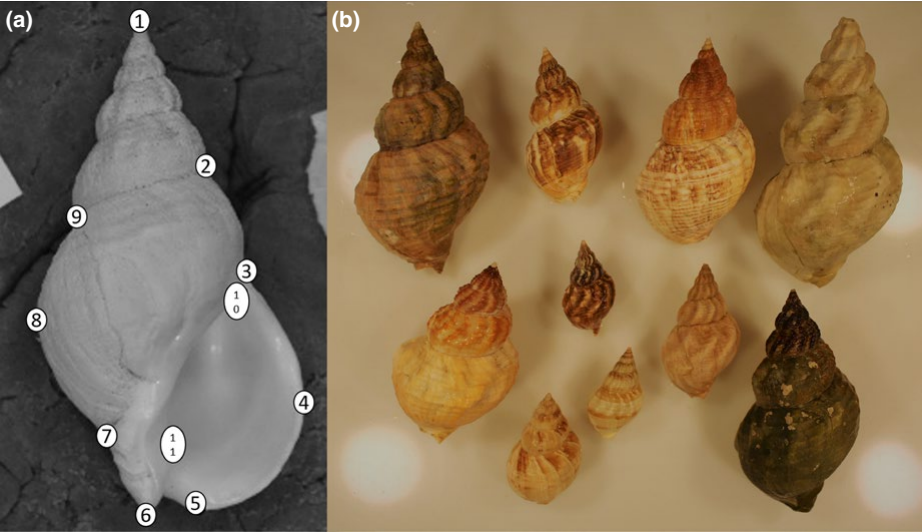
(a) The eleven landmarks from Mariani et al. (2012) digitized in the geometric morphometric analysis of shell shape of the common whelk. (b) An example of the morphological and colour variation of the common whelk in Breiðafjörður Bay in Iceland

Each shell was photographed in a consistent orientation, the ventral surface of the shell facing up and the anteroposterior axis at a right horizontal angle. We digitized 11 landmarks (Figure 3a) as in Mariani et al. (2012) using the R‐package *geomorph* (Adams & Otárola‐Castillo, 2013). Each and every individual was digitized twice and repeatability estimated based on the intraclass correlation coefficient (Arnqvist & Mårtensson, 1998). As repeatability was 0.81, the mean shape from the two repeated measurements of each shell was used for further analysis.

Procrustes distances between landmarks were generated based on the landmark data with a Generalized Procrustes Analysis in the *geomorph* package (Adams & Otárola‐Castillo, 2013). A Principal Components Analysis (PCA) was then performed using the same package on the superimposed data to clarify the main components of the morphological variation. Two principal components were used for subsequent analyses of the shape patterns, together they accounted for 45% of the total shape variance.

### 2.4 Color analysis

Color variation in all 344 shells was analyzed based on categorical scores (Table 2). Color of shells was scored manually using a Munsell‐based color scale, ColorChecker Classic from X‐rite (http://xritephoto.com/colorchecker-classic). The shell color of the common whelk is very patchy with more than 90% of the shells displaying two or more colors (Figure 3b). Three categories were scored for each shell: the most predominant color on the shell surface as well as the second and third most predominant colors. This yielded 128 combinations. For simplicity the analysis was limited to the two most predominant colors and similar colors were combined, resulting in 29 color types (Table A1). Presence and color of spiral stripes were also noted. The color variation at each sampling site was summarized by calculating the Shannon diversity index using the package *vegan* (Oksanen et al., 2016) in R (R Core Team 2016).

### 2.5 Population differentiation and its relation to environmental variation

The differentiation among samples was analyzed based on both shape and color:

1. *Geometric morphometrics*: Shape variation, partitioned among and within sites, considering sex, longitude, depth, substrate type, and proportion of decollated shells as covariates, was analyzed with Procrustes ANOVA in *geomorph* (Adams & Otárola‐Castillo, 2013).
2. *Traditional morphometrics*: Shape variation between sites was tested with ANOVA and *post hoc* Tukey's HSD.
3. Differences in frequencies of color composition and stripes between sites were tested with Fisher's exact test. Variation in proportion of striped individuals was further analyzed among and within sites with regards to longitude, depth, and substrate type using generalized linear models.
4. The association of morphology and color with environmental variation and geographic distances between sites was tested with Mantel's tests, with 1,000 permutations, using the R‐package *vegan* (Oksanen et al., 2016). Euclidean distances were calculated for the environmental variables as well as the morphology, while differences in color composition between sites were summarized with the Bray–Curtis distance (Oksanen, 2015).

## 3 RESULTS

### 3.1 Shell morphology, location, and environmental variables

**Figure 4:**
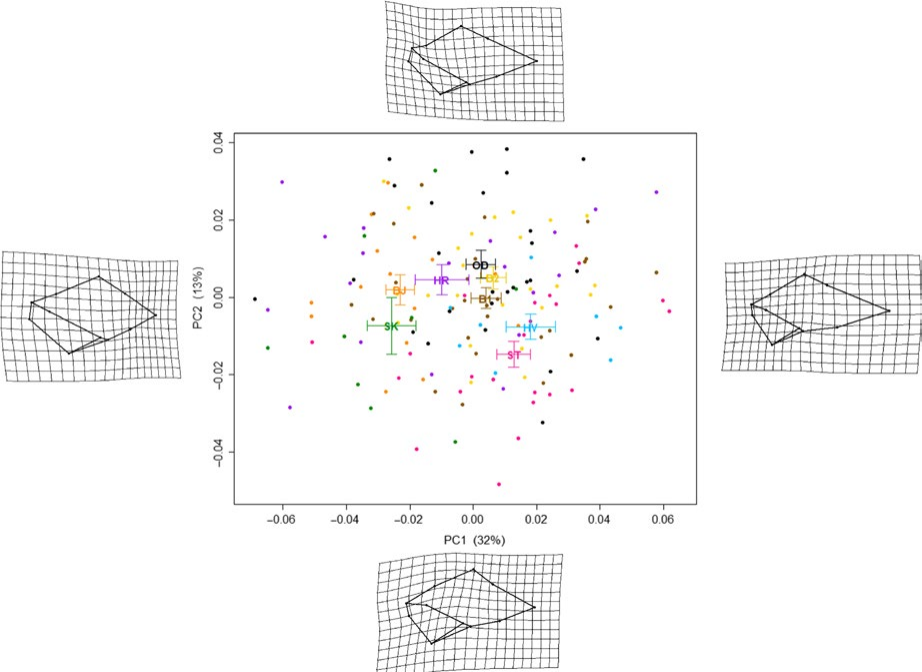
Shell shape variation of common whelk in Breiðafjörður Bay within and between sample sites. Percentages given in brackets refer to the proportion of the overall variation explained by the PC‐axis. Bars indicate one standard error and site codes are plotted at average PC values of each site. The transformation grids show the extreme shell shapes along the principal components. Procrustes ANOVA of variation in shell shape revealed a significant effect of both site and sex (see Table 3). Mean PC1 and PC2 values for male and female whelks were (−0.0157, −0.0011) and (0.0079, 0.0005), respectively

The first principal component of the landmark data explained 32% of the total shape variation and reflected the change in shell shape from rotund to elongate, with special emphasis on the ratio of the spire to the body whorl, as well as from an elongate aperture to a rounder aperture (Figure 4). The second principal component explained 13% of the variation and reflected the shape of the siphon, from slim to short and stubby.

A Procrustes ANOVA based on the results from the geometric morphometrics showed that there were significant differences in shape between sites and sexes (Table 3a). The shape of female whelks was significantly different from that of male whelks, with females having in general more elongate shells and a stubbier siphon than the males (*Procrustes ANOVA; F* = 9.49, *p* = .001). The mean values and low standard errors of PC1 and PC2 at each station (Figure 4) demonstrate that the groups are clearly distinguished along the two axes. Partitioning of the variation in shape between sites into sex, longitude, depth, substrate, and ratio of decollated shells using Procrustes ANOVA showed that the variables are all associated with shell shape (Table 3a). All predictor variables were significant (Table 3a) but longitude had the highest *R* ^2^ value (*R* ^2^ = .063) and thus explains more of the variation in shape than the other variables. However, longitude and depth were highly correlated (*r* = .68, *p* = .063) and even though the correlation was not significant it is still high enough for the two variables to affect each other in the model.

**Table 3:** Partitioning of the variation in shell traits with respect to sex, depth, substrate, longitude, and proportion of decollated shells. (a) Procrustes ANOVA of variation in shell shape, *R* ^2^ presents the proportion of variation explained by the variable model. (b) Proportion of striped individuals analyzed with logistic regression. AIC is the Aikaike criterion

Model	Predictor variables	Coefficient	Test statistic	Model evaluation
(a) Shell shape		*Z*	*F*	*R* ^2^
1	Sex	4.91	11.54**	.059
1	Site	8.47	5.51**	.198
1	Decollated	2.40	2.04**	.010
2	Sex	4.78	10.77***	.059
2	Longitude	5.27	11.51***	.063
2	Depth	2.19	2.35**	.013
2	Substrate	4.80	8.17***	.045
2	Decollated	2.38	2.25**	.012
(b) Prop. striped		Slope	*Z*	AIC
1	Depth	0.018	3.447***	347.93
2	Longitude	1.464	3.859***	343.87

Degrees of freedom were 1 (Sex) and 7 (Site) in model *a1* but 1 for each of the variables in the regression models. For more details see Table A2.

*p < .05, **.05 > *p* > 0.01, ***p < .01.

**Figure 5:**
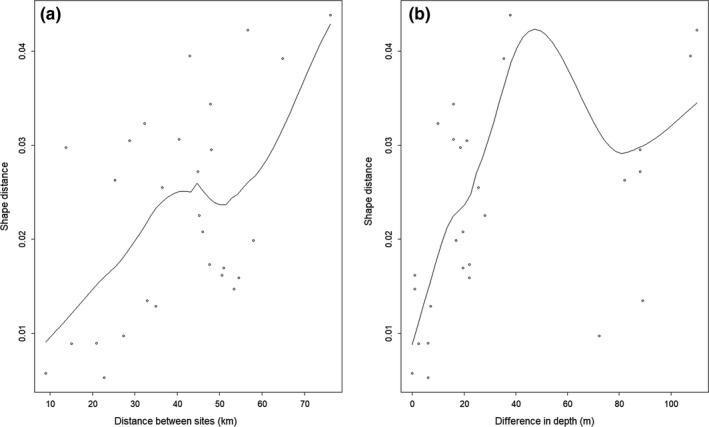
(a) Association of morphological and geographical distances of common whelk in Breiðafjörður Bay. (b) Association of morphological distances and difference in depth between sites. The morphological distances are Euclidean shape distances between the samples based on PC1 and PC2 from the geometric morphometrics. A smoothing curve generated by LOESS was fitted to the data

The geographical and spatial pattern of the samples in Breiðafjörður is reflected in the mean shape of the whelk, with the means aligning on the PC1 axis in strong correlation with the degree of longitude of their origin (Figure 4). The shape of the shell tends to go from elongate to round in an east‐to‐west direction, which corresponds to the inner and outer bay, respectively. Shell shape from elongate to round follows an increase in depth as well as a shift from hard substrate to a substrate with smaller particle size, such as sand and mud. Morphometric differentiation between sites increased with geographic distance (Figure 5a, Table 4, Mantel's *r* = .5274, *p* < .01) and with increase in depth (Figure 5b, Table 4, Mantel's *r* = .4427, *p* < .01) but was independent of differences in substrate between sites. Decollated shells were the most prevalent at each of the extreme sites, SK (0.67) and HV (0.50), but in general the proportion of decollated shells ranged from 0.11 to 0.35. There was a slight but significant effect of decollation on shape variation and possibly the analytical resolution of the ratio of the spire to the body whorl was affected by including decollated shells in the samples.

**Table 4:** Correlations of distances in shape and color diversity, with geographic distances, separation in depth and in ranking of substrate coarseness

	Shape	Color
Shape	–	–
Space	**0.5274** ***	0.1983
Depth	**0.4427** ***	0.2226
Substrate	0.3210	‐0.1430

Numbers in bold indicate significant correlation estimated with a Mantel test, 1,000 permutations.

*p < .05, **.05 > *p* > .01, ***p < .01.

**Figure 6:**
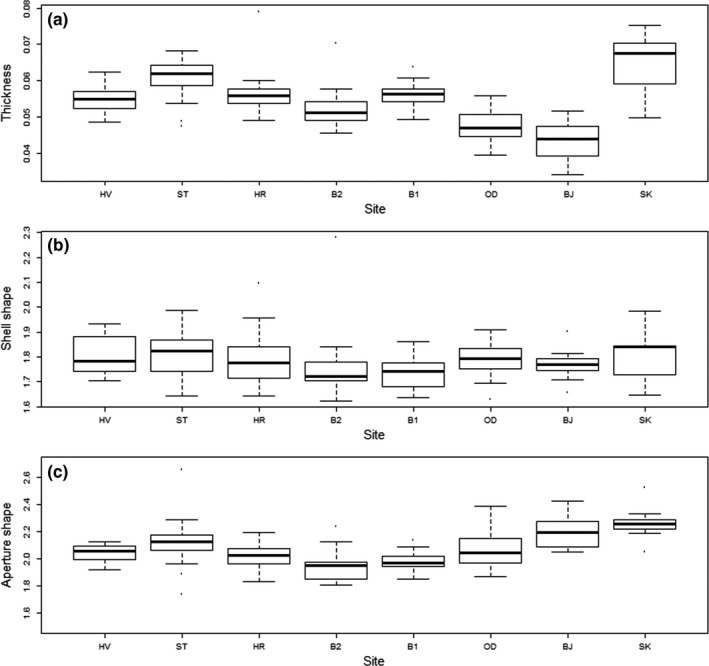
Traditional shell morphometrics of the common whelk at sample sites in Breiðafjörður Bay, ordered from east to west (see Table 1). (a) Thickness: √(Shell weight)/shell height. (b) Shell shape: Shell height/shell width. (c) Aperture shape: Aperture height/aperture width

Shell thickness and aperture shape, based on traditional morphometrics, varied between sites while there was no significant difference in shell shape (*thickness: F* = 36.47, *p* < .0001; *shape: F* = 1.87, *p* = .079; *aperture shape: F* = 9.16, *p* < .0001, Figure 6). In general, whelk shells in the outer part of Breiðafjörður were thinner than the shells in the inner part. This was confirmed by results from *post hoc* Tukey test where OD and BJ were significantly thinner than whelk shells at other sites in the inner part of the bay (Table A3). However, whelks at SK, the westernmost site, stood out from the general trend, being the thickest and most variable of all the whelk groups sampled in the study (Table A3). Aperture shape became increasingly elongate from east to west in the bay (Figure 6), but the trend was not as clear as for the thickness, as results from Tukey HSD showed that the SK and BJ are significantly different from the central sites in the bay (HR, B1, and B2), but not from HV and ST (the two eastern most sites; Table A4).

Principal component analysis of the three shape variables from the traditional morphometrics (thickness, shell shape, and aperture shape) displayed a similar spatial pattern to the landmark analyses (Figure A1), with the sites aligning along PC1 in order of increasing longitude, out the bay, except for HV and ST. The sampled populations could also be clearly defined based on these measurements, even though there was more variance in shell shape in each population than in those defined based on geometric morphometrics.

### 3.2 Color variation, location, and environmental variables

**Figure 7:**
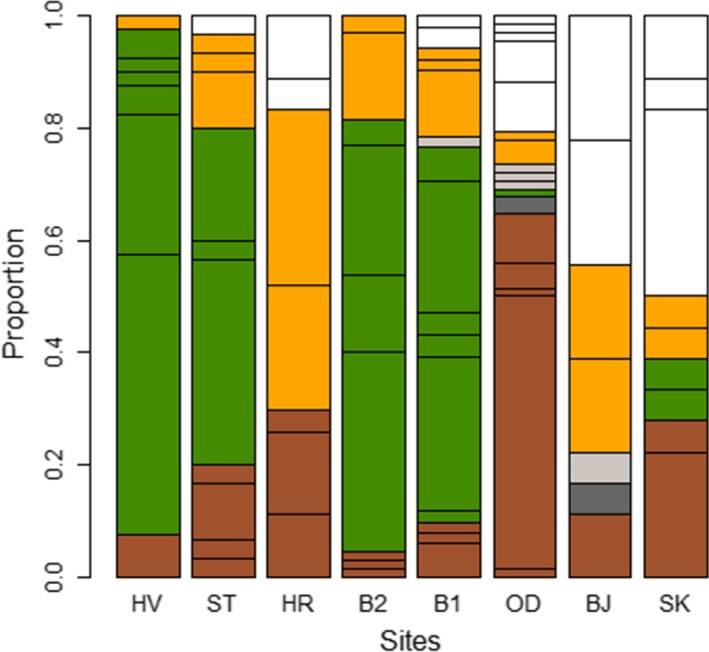
Shell colour types of the common whelk at sample sites, ordered from east to west, in Breiðafjörður Bay. Brownish, dark‐grey, greenish, grey, orange and whitish colour‐types are displayed in their respective colours. Horizontal lines within colour show more detailed colour within each class of colour types

A significant difference was observed in the frequency of color types between sites (Fisher's exact test: *p*‐value = .0005). Greenish color types were the most predominant shell color types at four sites: B1, B2, HV, and ST, while orange and whitish color types were the most predominant at the other sites (Figure 7, Table A1). As the greenish color is likely the result of algae or cyanobacteria residing in the shell (Hollyman, 2017), we also tested for difference in color‐type frequency excluding the sites where the greenish color type was predominant. There was still a significant difference in color‐type frequencies (Fisher's exact test: *p*‐value = .0005). Shannon diversity of color types at each site ranged from 1.46 at HV to 2.28 at B1. Differentiation in color composition between sites did not increase with geographic distance between sites, or with increased difference in depth or substrate (Table 4).

**Figure 8:**
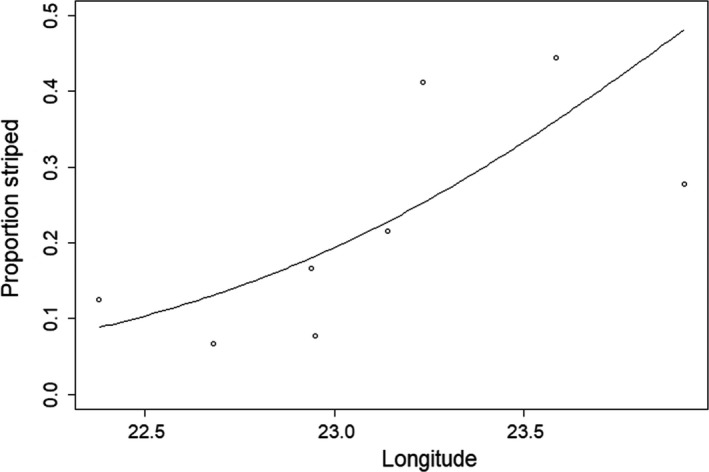
Proportion of striped individuals at the sample sites as a function of longitude. The curved line presents the prediction from a generalized linear model (See Table 3)

Striped individuals were found at all sites (Table 2) with a significant difference in the proportion of individuals between sites (χ^2^ = 35.88, *df* = 7, *p*‐value = 7.64 × 10^−06^). In all cases the stripes were brownish in coloring. The highest proportion of striped individuals was found at BJ while the fewest were found at B2. The proportion of striped individuals increased from the inner to the outer part of the bay, and also with increased depth (Table 3b). Based on the Aikaike criteria, longitude was the most significant predictor variable (Table 3b, Figure 8).

## 4 DISCUSSION

The common whelk in Breiðafjörður Bay show high morphological diversity with clear differences both in shape and color throughout the bay. Whelk populations were clearly spatially differentiated, which corroborates the results of previous studies on the species in the bay (Gunnarsson & Einarsson, 1995; Magnúsdóttir, 2010; Woods & Jonasson, 2017) and indicates limited demographic connectivity between populations. Phenotypic variation (thickness, shell shape, and color diversity) displayed a distinct pattern from east to west. In the innermost part, whelk shells were thick and elongate, had a round aperture, and were less diverse in color. Outward in the bay the shells gradually became thinner with a rounder shell, had a more elongate aperture, and displayed more color diversity. Whelk shells were also lighter in shell color and more likely to be striped further outward in the bay. Differences between genders were in line with previous studies (Kenchington & Glass, 1996; Ten Hallers‐Tjabbes, 1979; Valentinsson, 2002) where females had higher or more elongate shells.

The degree of differentiation in the whelk shell traits reflected geographic distances, suggesting that demographic connectivity between populations is restricted. Populations closer to each other were more similar in shell traits than populations further away, which is consistent with our previous study on genetic population structure within Breiðafjörður Bay (Pálsson et al., 2014). Therein, genetic differentiation followed an isolation‐by‐distance model and samples separated by 13 km showed a significant degree of genetic differentiation. Dispersal over short distances between adjacent populations was an expected pattern in the context of life‐history traits that may limit dispersal, in particular direct development and a relatively sedentary adult life style (Himmelman, 1988; Himmelman & Hamel, 1993; Jalbert, Himmelman, Béland, & Thomas, 1989).

Studies by Weetman et al. (2006) on neutral genetic variation of the common whelk showed that migration rates on the British west coast were asymmetric. Inshore to offshore migration was favored, with lower diversity and higher differentiation more evident in inlet populations than in offshore populations. Higher diversity in western sites at greater depth in Breiðafjörður may indicate an inshore–offshore migration pattern as well. Whelk density is highest in the inner part of the bay so competition there could raise emigration rates to the outer areas, as proposed by Weetman et al. (2006) for whelks on the British west coast. Our previous work on genetic variation of whelk within the bay (Pálsson et al., 2014) does not provide information about the direction of the migration as it was only based on three sites in the center of the bay, HR and OD plus an additional one (Hempill), approximately 13 km northwest of HR.

In general, there was a distinct trend of decreasing shell thickness from east to west, with the exception of the two marginal sites (HV to the east and SK to the west). However, longitude was nominally positively correlated with substrate and depth and these variables influence the benthic community composition, which again may indirectly affect the morphology of the whelk via changes in predatory species composition (Rochette & Himmelman, 1996) or other species in the ecosystem assemblage that may provide cover from predators, such as macroalgae. However, the correlation between longitude and depth makes it hard to disentangle these variables but both seem to have an effect on the shell shape. With regards to the marginal sites, the shells at HV are less dense than at its adjacent site ST, possibly because of the prevalence of shell boring organisms such as cyanobacteria or algae (Hollyman, 2017). The large variance in thickness of whelks at SK could be related to a sharp change in environmental variables at the outskirts of Breiðafjörður, but it is not possible to draw any concrete conclusions due to lack of environmental data.

In the inner part of Breiðafjörður, the prevalence of thick shells with elongate spires and small round apertures could be linked to crab predation (Bourdeau & Johansson, 2012). Spider crab (*Hyas araneus*), green crab (*Carcinus maenas*), and the recent invader, the Atlantic rock crab (*Cancer irroratus*; Gíslason et al., 2013), are found in Breiðafjörður and are known to feed on whelks. All three crab species prefer shallower areas to deeper ones (Klassen & Locke, 2007; Vargo & Sastry, 1977; Walther, Sartoris, Bock, & Pörtner, 2009), so their effect would be more pronounced in the inner part of the bay.

The traditional morphometrics yielded similar results to the geometric morphometrics; groups were clearly separated and showed a clear east–west trend, aside from the two sites at the opposite ends of the range at HV and SK. The discordance between the two morphometric methodologies could result from different shell variables being assessed in the two methods.

Although the common whelk is widely distributed throughout the N‐Atlantic (Gendron, 1992; Golikov, 1968), Breiðafjörður Bay is the only known area where the species exhibits such a wide range of color variants over small geographic distances. Color diversity and frequency of color types differed significantly between sites even though there was considerable within‐site diversity. This variation among sites could reflect random fluctuations in color frequencies within the separate populations and thus be an indicator of lower demographic connectivity. High localized color diversity could also reflect a functional role as camouflage in a heterogeneous environment such as Breiðafjörður Bay (Breiðafjörður‐Conservation 2014; Stevens, Lown, & Wood, 2014) or partly reflect the different environmental settings, for example, influences from algae.

In general, the surface waters and pelagic zone within Breiðafjörður are considered to be relatively well mixed with regards to nutrients, salinity, and temperature (MRI, 2016), however, there could be a gradient effect on the surface waters from freshwater brought into the bay by the clockwise coastal current (Logemann, Ólafsson, Snorrason, Valdimarsson, & Marteinsdóttir, 2013). The hydrological conditions of the benthic communities in Breiðafjörður have not been studied so far, and to make concrete conclusions on the effects on shell morphology and color of predatory benthic gastropods, further studies are necessary.

Characterized by a large number of islands and widespread intertidal and shallow subtidal areas, the bay is one of the most biodiverse areas in Icelandic waters (Breiðafjörður‐Conservation 2014) and has the highest density of whelk around Iceland (Gunnarsson & Einarsson, 1995). In this region, whelk are part of the diets of a variety of animal such as eiders, cod, wolffish, starfish, and crabs, and in turn whelk have the opportunity for a very varied diet. This could have indirect or direct effects on their shell color (Lindberg & Pearse, 1990; Manríquez, Lagos, Jara, & Castilla, 2009). For example, some marine gastropods have a similar shell color lightness to the color of their prey; an adaptive camouflage response to avoid predators or an indicator of phenotypic plasticity (De Bruyn & Gosselin, 2014; Manríquez et al., 2009; Stevens, 2016).

Whelk in Breiðafjörður Bay exhibited fine‐scale patterns of spatial variation in the various independent shell traits quantified in this study, where divergence increased with geographic distances. This is a strong indicator that demographic connectivity of the common whelk is limited, even in an area as small as Breiðafjörður Bay, and is in accordance with observed genetic differentiation over short distances within the bay (Pálsson et al., 2014). The environmental gradient in the bay reflects the phenotypic gradient of the common whelk. This relationship may indicate that the local environmental conditions have shaped the morphological variation, resulting in distinct ecotypes showing ecological segregation (Johannesson, 2015). Whether such a relationship between morphological variation and depth is found in other areas warrants further studies and may indicate a selective cline. Evaluation of the impact of environmental effects on the morphological variation by applying common garden experiments and assessing the underlying genetic variability of the shell traits is currently being conducted.

## CONFLICT OF INTEREST

None Declared.

## AUTHOR CONTRIBUTIONS

All authors participated in and designed the study. Field work was done by HM, KMW, and ZOJ while HM did the measurements and analyzed the data together with SP. HM and SP wrote the manuscript which was edited by KMW, ZOJ, and EBÖ.
