# Supplementary figures and images for: Shell morphology and color of the subtidal whelk Buccinum undatum exhibit fine‐scaled spatial patterns
Source: Ecol Evol. 2018 Apr 10;8(9):4552–63. doi: 10.1002/ece3.4015 (PMC5938454; doi:10.1002/ece3.4015)

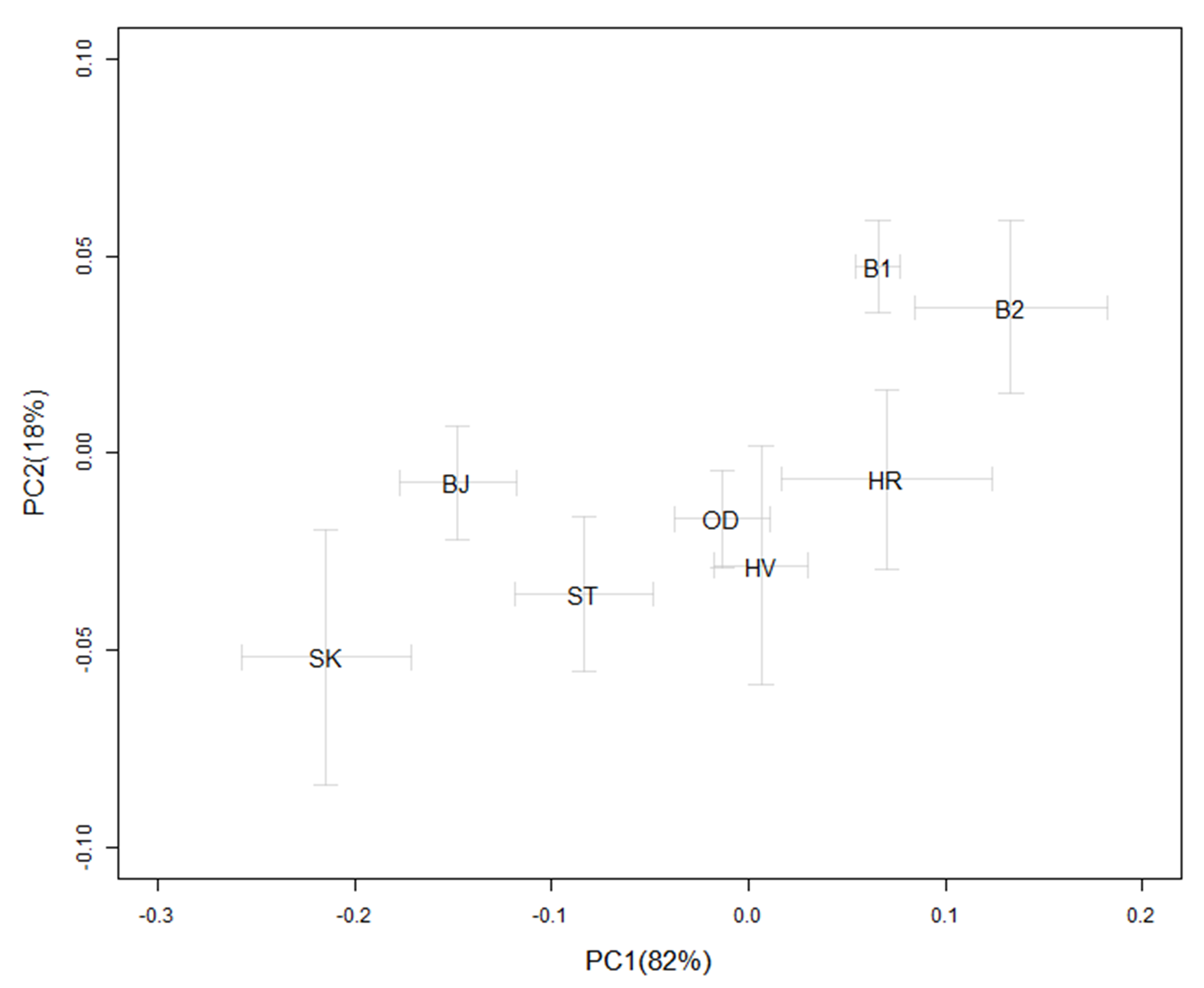

Supplement: Supplementary file 1 [file ECE3-8-4552-s001.tif]
