# Supplementary material for: Shell morphology and color of the subtidal whelk Buccinum undatum exhibit fine‐scaled spatial patterns
Source: Ecol Evol. 2018 Apr 10;8(9):4552–63. doi: 10.1002/ece3.4015 (PMC5938454; doi:10.1002/ece3.4015)
Supplement: Supplementary file 2 [file ECE3-8-4552-s002.docx]

**APPENDIX**

**Table A1.** Common whelk colour-type combinations at the sample sites in Breiðafjörður Bay.

|  | BJ | B1 | B2 | HR | HV | OD | SK | ST |
| --- | --- | --- | --- | --- | --- | --- | --- | --- |
| Brownish_Blue | 0 | 0 | 0 | 0 | 0 | 1 | 0 | 0 |
| Brownish_Brownish | 2 | 3 | 1 | 6 | 0 | 33 | 4 | 1 |
| Brownish_Greenish | 0 | 0 | 1 | 0 | 3 | 0 | 0 | 1 |
| Brownish_Grey | 0 | 0 | 0 | 0 | 0 | 1 | 0 | 0 |
| Brownish_Orange | 0 | 1 | 1 | 8 | 0 | 3 | 1 | 3 |
| Brownish_Whitish | 0 | 1 | 0 | 2 | 0 | 6 | 0 | 1 |
| Darkgrey_Brownish | 0 | 0 | 0 | 0 | 0 | 2 | 0 | 0 |
| Darkgrey_Whitish | 1 | 0 | 0 | 0 | 0 | 0 | 0 | 0 |
| Greenish_Blue | 0 | 1 | 0 | 0 | 0 | 0 | 0 | 0 |
| Greenish_Brownish | 0 | 14 | 23 | 0 | 20 | 0 | 0 | 11 |
| Greenish_Greenish | 0 | 2 | 9 | 0 | 10 | 0 | 1 | 1 |
| Greenish_Grey | 0 | 2 | 0 | 0 | 2 | 1 | 0 | 0 |
| Greenish_Orange | 0 | 12 | 15 | 0 | 1 | 0 | 0 | 6 |
| Greenish_ModRed | 0 | 0 | 0 | 0 | 1 | 0 | 0 | 0 |
| Greenish_Whitish | 0 | 3 | 3 | 0 | 2 | 0 | 1 | 0 |
| Grey_Brownish | 0 | 0 | 0 | 0 | 0 | 1 | 0 | 0 |
| Grey_Grey | 0 | 0 | 0 | 0 | 0 | 1 | 0 | 0 |
| Grey_Orange | 0 | 1 | 0 | 0 | 0 | 0 | 0 | 0 |
| Grey_Whitish | 1 | 0 | 0 | 0 | 0 | 1 | 0 | 0 |
| Orange_Brownish | 3 | 6 | 10 | 12 | 1 | 3 | 1 | 3 |
| Orange_Greenish | 0 | 1 | 0 | 0 | 0 | 0 | 0 | 1 |
| Orange_Orange | 3 | 1 | 2 | 17 | 0 | 0 | 1 | 1 |
| Orange_Whitish | 0 | 0 | 0 | 0 | 0 | 1 | 0 | 0 |
| Whitish_Brownish | 4 | 0 | 0 | 3 | 0 | 6 | 6 | 0 |
| Whitish_Greenish | 0 | 2 | 0 | 0 | 0 | 0 | 0 | 0 |
| Whitish_Grey | 0 | 0 | 0 | 0 | 0 | 5 | 1 | 0 |
| Whitish_Orange | 0 | 0 | 0 | 6 | 0 | 1 | 0 | 1 |
| Whitish_Purple | 0 | 0 | 0 | 0 | 0 | 1 | 0 | 0 |
| Whitish_Whitish | 4 | 1 | 0 | 0 | 0 | 1 | 2 | 0 |

**Table A2.** Detailed information for the models presented in Table 3. Partitioning of the variation in shell traits with respect to site, sex, depth, substrate, longitude and proportion of decollated shells. (a) Procrustes ANOVA of variation in shell shape. (b) Proportion of striped individuals analysed with logistic regression. * p < 0.05, ** 0.05 > p > 0.01, ***p<0.01

| Model | Predictor variables |  |  |  | Coef | Test statistic | Model evaluation |
| --- | --- | --- | --- | --- | --- | --- | --- |
| *(a) Shell shape* |  | Df | SS | MS | Z | F | R^2^ |
| 1 | Sex | 1 | 0.02108 | 0.0210764 | 4.9096 | 11.5418** | 0.059141 |
| 1 | Site | 7 | 0.07044 | 0.0100625 | 8.4725 | 5.5104** | 0.197651 |
| 1 | Decollated | 1 | 0.00373 | 0.0037281 | 2.3978 | 2.0416** | 0.010461 |
| 1 | Residuals | 143 | 0.26113 | 0.0018261 |  |  |  |
|  | Total | 152 | 0.35637 |  |  |  |  |
| 2 | Sex | 1 | 0.02108 | 0.0210764 | 4.7779 | 10.7663**** | 0.059141 |
| 2 | Longitude | 1 | 0.02252 | 0.0225244 | 5.2667 | 11.5060*** | 0.063205 |
| 2 | Depth | 1 | 0.00460 | 0.0046006 | 2.1867 | 2.3501** | 0.012910 |
| 2 | Substrate | 1 | 0.01600 | 0.0160033 | 4.7964 | 8.1748*** | 0.044906 |
| 2 | Decollated | 1 | 0.00440 | 0.0043969 | 2.3762 | 2.2460** | 0.012338 |
| 2 | Residuals | 147 | 0.30095 | 0.0020334 |  |  |  |
|  | Total | 152 | 0.35637 |  |  |  |  |
| *(b) Prop. striped* |  |  | a | Z | B | z | AIC |
| 1 | Intercept |  | -2.045 | -7.922*** |  |  |  |
| 1 | Depth |  |  |  | 0.018 | 3.447*** | 347.93 |
|  |  |  |  |  |  |  |  |
| 2 | Intercept |  | -35.108 | -4.001*** |  |  |  |
| 2 | Longitude |  |  |  | 1.464 | 3.859*** | 343.87 |

**Table A3.** Results from *post-hoc* Tukey‘s HSD test of difference in thickness between sites. Significant differences are in highlighted in bold.

|  | **HV** | **ST** | **HR** | **B2** | **B1** | **OD** | **BJ** | **SK** |
| --- | --- | --- | --- | --- | --- | --- | --- | --- |
| **HV** |  | 0.1032 | 0.9943 | 0.8970 | 1.0000 | **0.0107** | **1.1*10^-5^** | **0.0075** |
| **ST** |  |  | **0.1444** | **0.0000** | **0.0187** | **0.0000** | **0.0000** | 0.7272 |
| **HR** |  |  |  | 0.1058 | 1.0000 | **0.0000** | **0.0000** | **0.0092** |
| **B2** |  |  |  |  | 0.1670 | **0.0308** | **6*10^-6^** | **0.0000** |
| **B1** |  |  |  |  |  | **0.0000** | **0.0000** | **0.0012** |
| **OD** |  |  |  |  |  |  | 0.12 | **0.0000** |
| **BJ** |  |  |  |  |  |  |  | **0.0000** |
| **SK** |  |  |  |  |  |  |  |  |

**Table A4.** Results from *post-hoc* Tukey‘s HSD test of difference in aperture shape (traditional morphometrics) between sites. Significant differences are in highlighted in bold.

|  | **HV** | **ST** | **HR** | **B2** | **B1** | **OD** | **BJ** | **SK** |
| --- | --- | --- | --- | --- | --- | --- | --- | --- |
| **HV** |  | 0.8620 | 0.9670 | 0.4501 | 0.9660 | 1.0000 | 0.3715 | 0.0833 |
| **ST** |  |  | **0.0378** | **9.45*10^-5^** | **0.0133** | 0.7509 | 0.9424 | 0.4089 |
| **HR** |  |  |  | 0.8653 | 1.0000 | 0.6399 | **0.0029** | **0.0004** |
| **B2** |  |  |  |  | 0.8050 | **0.0191** | **6*10^-6^** | **1.5*10^-6^** |
| **B1** |  |  |  |  |  | 0.4937 | **8*10^-4^** | **0.0001** |
| **OD** |  |  |  |  |  |  | 0.1642 | **0.0230** |
| **BJ** |  |  |  |  |  |  |  | 0.9632 |
| **SK** |  |  |  |  |  |  |  |  |
